# The gap between tap water beliefs and preference for drinking from the tap: a cross-sectional study in Virginia, USA

**DOI:** 10.1017/S1368980025101365

**Published:** 2025-10-20

**Authors:** Jasmine H. Kaidbey, Uriyoán Colón-Ramos, Hannah Robbins Bruce, Allison C. Sylvetsky

**Affiliations:** 1 Department of Exercise and Nutrition Sciences, Milken Institute School of Public Health, The George Washington University, 950 New Hampshire Avenue NW, Suite 200, Washington, DC 20037, USA; 2 Department of Global Health, Milken Institute School of Public Health, The George Washington University, 950 New Hampshire Avenue NW, Suite 500, Washington, DC 20037, USA; 3 Virginia Foundation for Healthy Youth, 701 E. Franklin Street, Suite 500, Richmond, VA 23219, USA

**Keywords:** Tap water safety, Drinking tap water, Bottled water, Trust in drinking water, Trust in water utility

## Abstract

**Objective:**

Half of people living in the USA do not consume tap water. Surveys have assessed perceptions of water and water utilities, but less is known about how these perceptions relate to the preference for tap or bottled water. The present analysis examined whether beliefs about tap water and the water utility were associated with drinking water preferences.

**Design:**

In a cross-sectional survey, six water beliefs were measured: trust in tap water, the water utility, and the local government; perceived safety and quality of tap water; and awareness that the water utility frequently tests tap water. Regression models adjusted for sociodemographic characteristics were used to estimate the odds of preferring tap over bottled water dependent on respondents’ beliefs about their tap water.

**Setting:**

Virginia, USA.

**Participants:**

Adults aged 18 years and older (*n* 808).

**Results:**

More than two-thirds of respondents had positive beliefs about their tap water, but only 54 % reported tap as their preferred drinking water source. All water beliefs, except for awareness of the frequency of water testing, were associated with higher odds of preferring tap water over bottled (adjusted OR range: 1·56–3·2).

**Conclusions:**

Our findings suggest that favourable tap water beliefs may be necessary, but not sufficient, to motivate people to drink from the tap. There remains a critical need for future research to bridge the gap between tap water perceptions and consumption, which should include enhancing the trustworthiness of tap water and the water utility as well as improving consumers’ perceptions of water quality and safety.

Sugar-sweetened beverages are the predominant source of added sugar in the American diet and often displace intake of healthier beverages^(1)^. Added sugar intake is robustly associated with obesity, tooth decay, type 2 diabetes and other cardiometabolic risk factors^(2–4)^, and the Dietary Guidelines for Americans recommend limiting added sugars to no more than 10 % of daily calories and encourage replacement of sugar-sweetened beverages with unsweetened alternatives, such as plain water^(5)^.

From a cardiometabolic health perspective, consumption of any form of plain water is preferable to consuming a sugar-sweetened beverage; however, bottled water consumption has profound disadvantages for the environment. Briefly, if plastic bottles are littered or not properly contained in the landfill, they turn into debris that can disturb marine habitats, and their degradation in landfills releases microplastics into the ground, which can contaminate water sources^(6,7)^. Even in cases where consumers discard bottles for recycling, a large percentage of the bottles cannot be recycled^(8)^. Although the more recent introduction of canned and boxed water products may be favourable to plastic bottles, the production and transportation of all of these products require more resources compared to drinking water directly from the tap^(9)^.

The decision to drink water directly from the faucet, to filter water, or to consume bottled water, is a complex behaviour driven by several interconnected factors and historical experiences. It has been posited that individuals choose their drinking water source depending on their (1) trust in the safety of water, (2) perceived risk and severity of an adverse event following the use of the water, (3) the salience of past events involving water, (4) sensory evaluations of the water such as its taste, odour and appearance^(10)^, and (5) willingness to pay for alternatives (e.g. bottled water or filtration systems)^(8)^. Surveys have assessed Americans’ attitudes and knowledge of their water utility^(11,12)^, but little is known about how these perceptions of the water utility relate to drinking water preferences, with the exception of one survey in Virginia that found that individuals who drink exclusively from the faucet had higher levels of trust in the water utility compared to individuals who drink exclusively bottled water^(10)^. This supports the notion that trust in the utility is positively associated with drinking from the tap^(13)^ and raises the question about which specific features of the water utility – such as awareness of routine tap water testing – may shape water preferences. The purpose of this cross-sectional analysis is to examine whether perceptions of water and the water utility are associated with the preferred water source among adults in Virginia.

## Methods

### Data source

The Water Beliefs Survey was designed to assess Virginians’ perceptions about their water and identify drinking water preferences. It was developed by an interdisciplinary team at the Virginia Foundation for Healthy Youth and Virginia Health Catalyst working with a broad taskforce to ensure equitable access to safe and affordable drinking water that is trusted and preferred by all Virginians. The goal of the survey was to gauge the current perceptions in order to inform intervention strategies to increase tap water consumption. Market Decisions Research, a research company, recruited participants by address-based, mailed survey invitations and by an online panel assembled by Voxco, a market research company. The survey was administered in December 2022 and completed by 1576 Virginia residents aged 18 years and older, using an online survey tool (Voxco). Individuals with private wells were oversampled to address a separate research question; the present analysis only includes participants with a water utility, consistent with 90 % of the US population^(14)^. The 743 participants who used private wells or rainwater harvesting as their source of drinking water were excluded because they did not respond to survey questions about the water utility. An additional twenty-four participants were excluded due to having incomplete data for any of the survey items used in this analysis (described below). This study was determined to be research that is exempt from Institutional Review Board review under Department of Health and Human Services regulatory category 4.

### The Water Beliefs Survey items

The Water Beliefs Survey included questions about demographic characteristics, water perceptions and water intake preferences.

#### Demographic characteristics

Self-reported demographic characteristics included in this analysis were selected based on hypothesised or previously demonstrated associations with water intake. These were participants’ age, sex, race/ethnicity, education level, housing status and region of residence within the state of Virginia and are presented in Table 1. Education, rather than income, was selected as a socio-economic indicator because it had a stronger association to drinking water preference.

**Table 1.** Self-reported demographic characteristics of 808 adults residing across the state of Virginia who participated in the Water Beliefs Survey

Characteristic	*n*	%
Race/ethnicity		
White	582	72·0
Black	158	19·6
Asian	28	3·5
Hispanic or Latino	14	1·7
Native American/Alaska Native/Native Hawaiian/Pacific Islander	3	0·4
Other/Prefer not to answer	23	2·8
Education		
High school diploma or less	264	32·7
Some college or associate’s degree	147	18·2
College graduate or more	397	49·1
Gender		
Male	300	37·1
Female	481	59·5
Other	27	3·3
Age^*^	46·1	17·1
Region of Virginia		
Central	145	17·9
Eastern	214	26·5
Northern	166	20·5
Northwest	118	14·6
Southwest	165	20·4
Housing status		
Homeowner	492	60·2
Renter	285	34·9
Other/Prefer not to answer	40	4·9
Household income		
Under $24 000	132	16·3
$24 000–$49 999	184	22·8
$50 000–$74 999	176	21·8
$75 000–$99 999	96	11·9
$100 000 or more	220	27·2

* Mean and standard deviation.

#### Water beliefs

We included six survey questions that aligned with previously posited determinants of tap water intake. To assess trust in water, three questions were used: (1) ‘How much, if at all, do you trust the water at your faucet?’, (2) ‘How much, if at all, do you trust your water utility company?’ and (3) participants were asked if they considered the local government a trusted source for information about water (binary). Perceived quality and safety of the water were assessed by asking: (4) ‘How would you rate the quality of the water at your faucet?’ and (5) ‘In your view, how safe or unsafe is the water at your faucet?’. Finally, awareness of water testing was probed by the following question: (6) ‘How aware are you, if at all, that utilities frequently test your water?’. Response options to all survey questions are displayed in Table 2.

**Table 2.** Response frequencies for survey questions about water perceptions, stratified by preferred source of drinking water, among adults in Virginia surveyed in the Water Beliefs Survey

	Preferred source of drinking water					
Water belief	Bottled (*n* 370)		Tap (*n* 438)		All (*n* 808)	
	*n*	%	*n*	%	*n*	%
Trust in water at the faucet						
None at all/Not too much	121	32·7	83	18·9	204	25·2
Don’t know/No opinion	26	7·0	34	7·8	60	7·4
Some	163	44·1	153	34·9	316	39·1
A lot	60	16·2	168	38·4	228	28·2
Trust in water utility						
None at all/Not too much	46	12·4	31	7·1	77	9·5
Don’t know/No opinion	54	14·6	50	11·4	104	12·9
Some	166	44·9	174	39·7	340	42·1
A lot	104	28·1	183	41·8	287	35·5
Rating of water quality						
Poor	27	7·3	19	4·3	46	5·7
Just fair	125	33·8	78	17·8	203	25·1
Good	173	46·8	228	52·1	401	49·6
Excellent	45	12·2	113	25·8	158	19·6
Safety of water at the faucet						
Very/Somewhat unsafe	66	17·8	49	11·2	115	14·2
I don’t know/No opinion	63	17·0	42	9·6	105	13·0
Somewhat safe	160	43·2	167	38·1	327	40·5
Very safe	81	21·9	180	41·1	261	32·3
Local government is a trusted source of information about water						
Yes	148	42·7	251	60·0	399	50·6
No	222	57·3	187	40·0	409	49·4
Awareness that water utility frequently tests water						
Not aware at all	63	17·0	57	13·0	120	14·9
Not very aware	91	24·6	112	25·6	203	25·1
Somewhat aware	131	35·4	156	35·6	287	35·5
Very aware	85	23·0	113	25·8	198	24·5
	Mean	sd	Mean	sd	Mean	sd
Belief score* (Mean, sd)	15·5	4·1	17·6	4·3	16·7	4·3

* Belief score is a composite of responses to the six water beliefs: trust in tap water, the water utility and the local government; perceived safety and quality of tap water; and awareness that the water utility frequently tests tap water.

#### Preferred source of drinking water

Preferred source of drinking water was determined using the survey item ‘What is your preferred source of drinking water?’. Those who reported preferring any tap water (i.e. directly from the faucet or from a filtering system) were compared to those who preferred bottled water.

### Descriptive statistics

Survey responses were summarised descriptively using frequencies (*n*) and percentages. For Likert scale responses, the two lowest response options were combined (i.e. ‘not at all’/‘not too much’ for trust and ‘very unsafe’/‘somewhat unsafe’ for safety). Contingency tables for each water belief and preferred drinking water source were tested for an association using a *χ*^2^ test.

### Multivariable statistics

Logistic regression was used to estimate the adjusted OR and 95 % CI of a preference for tap water consumption (filtered or unfiltered), compared to a preference for bottled water consumption. A total of seven multivariable models were conducted, as described below.

#### Water beliefs tested as individual, independent variables

Each of the six water beliefs was considered an independent variable to estimate the odds of preferring tap (filtered or unfiltered), compared to bottled, water. Water beliefs were analysed as multi-category factors, where response options (e.g. ‘a lot’) were compared to the lowest response option as the reference category (e.g. ‘not at all/not too much’).

#### Composite belief score

A belief score was created by assigning integer values to each of the six water belief survey items and summing the scores. Beliefs with Likert response options were assigned a 1 for the lowest option (i.e. ‘not at all/not too much’) and a 4 for the highest (‘a lot’). For the only belief with a binary response option – considering the government as a trusted source of information about water – a 1 was assigned for no and a 4 for yes. An internal consistency analysis was conducted, and the items demonstrated reliability (Cronbach’s α = 0·78). The belief score was then used as an independent variable to estimate the odds of preferring tap, compared to bottled, water.

A sensitivity analysis was conducted with the outcome of preferring unfiltered tap water, compared to the other two water sources (filtered and bottled water), for each multivariable model. All models were adjusted for the demographic covariates described above. Extreme observations, defined as those with a standardised residual greater than 3, were removed (*n* 1). Variance inflation factors were calculated to diagnose multicollinearity, but no variables with a variance inflation factor above 5 were detected. All analyses were performed using R Statistical Software^(15)^.

## Results

A total of 808 participants were included in this analysis (Table 1). Participants were mostly White (72 %), followed by Black (19·6 %), Asian (3·5 %), Hispanic/Latino (1·7 %) or Native/Pacific Islander (0·4 %). About one-third of the sample had educational attainment of a high school diploma or less, and about half had at least a college degree. More than half of the participants were female, homeowners, and had a household income of at least $50 000. About a quarter of participants were residents of eastern Virginia, with the remainder spread across northern, northwestern, central and southwestern regions of the state.

The most commonly preferred water source was bottled water (*n* 370), followed by filtered water (*n* 312). Only 126 participants (15·6 %) indicated that water directly from the faucet (i.e. unfiltered) was their preferred drinking water source. Response frequencies for water beliefs are shown in Table 2. The proportion of participants who had favourable responses (i.e. ratings above the midpoint of the Likert scale) was higher among those who reported a preference for tap water compared to those who preferred bottled water. The mean belief score was 16·7 (sd: 4·3) and ranged from 6 to 24 (the full range of possible values). All water beliefs, except for awareness of the frequency of water testing, were associated with preferred water source (all *P* < 0·001, see online supplementary material, Supplemental Table 1).

Although only 54·2 % indicated that tap water was their preferred source for drinking, most participants (67 % to 78 % depending on the question) indicated favourable water perceptions (e.g. selected ‘somewhat safe’, ‘some trust’, ‘good quality’, ‘somewhat aware’ or higher). Two-thirds of the participants indicated having some or a lot of trust in their tap water, 78 % had some or a lot of trust in their water utility, 69 % rated the quality of their water as good or excellent and 73 % considered their water somewhat or very safe. Approximately one-third trusted their tap water (*n* 228) and water utility a lot (*n* 291) and considered their water very safe (*n* 261). About a quarter of participants indicated being very aware that the water utility tests the water frequently (*n* 200) and 19·6 % rated the quality of their water as excellent. Approximately half (49 %) of the sample considered the government a trusted source for information about water.

In the multivariable models for each water belief except awareness of water testing, participants who selected the highest value (e.g. ‘excellent’ for quality, or ‘a lot’ for trust in the utility) had higher odds of preferring tap water, compared to the reference category (‘not at all/not too much’) (Table 3). The highest odds of preferring tap water were observed among those with the highest levels of trust in water at the faucet (OR: 3·20, 95 % CI: 2·06, 5·02, *P* < 0·001), followed by those who: rated their tap water quality as excellent (OR = 2·54, 95 % CI: 1·22, 5·36, *P* = 0·013), rated their tap water as very safe (OR = 2·32, 95 % CI: 1·42, 3·82, *P* < 0·001), had a lot of trust in the water utility (OR: 1·85, 95 % CI: 1·06, 3·28, *P* = 0·03) and considered the local government a trusted source for information (OR: 1·56, 95 % CI: 1·14, 2·12, *P* = 0·005). In the belief score model, each additional point was associated with 9 % higher odds of preferring tap compared to bottled water (OR: 1·09, 95 % CI: 1·05, 1·13, *P* < 0·001). Although the magnitude of the OR varied by model, the patterns seen across covariates remained constant (see online supplementary material, Supplemental Tables 2–8). Educational attainment was the most influential demographic characteristic associated with drinking water preferences: adults with a college education were about three times more likely to prefer drinking tap water, compared to those with a high school diploma or less. The direction and significance of all associations were preserved in the sensitivity analysis that tested for the odds of preferring unfiltered tap water compared to filtered or bottled water (see online supplementary material, Supplemental Table 9).

**Table 3.** Water beliefs association with preferred water source among adults in Virginia surveyed in the Water Beliefs Survey (*n* 808)

Multivariable model independent variable	Adjusted OR	95 % CI	*P*-value
How much, if at all, do you trust the water at your faucet?			
Not at all/Not too much	Reference		
No opinion/Don’t know	1·78	0·95, 3·39	0·07
Some	1·17	0·80, 1·72	0·4
A lot	3·20	2·06, 5·02	< 0·001
How would you rate the quality of the water at your faucet?			
Poor	Reference		
Just fair	0·78	0·39, 1·57	0·5
Good	1·63	0·85, 3·19	0·1
Excellent	2·54	1·22, 5·36	0·01
In your view, how safe or unsafe is the water at your faucet?			
Not safe at all/Not very safe	Reference		
Somewhat unsafe	0·95	0·53, 1·70	0·9
Somewhat safe	1·30	0·82, 2·06	0·3
Very safe	2·32	1·42, 3·82	< 0·001
How much, if at all, do you trust your water utility company?			
Not at all/Not too much	Reference		
No opinion/Don’t know	1·33	0·70, 2·55	0·4
Some	1·33	0·78, 2·31	0·3
A lot	1·86	1·06, 3·28	0·03
Local government is a trusted source of information (yes)	1·56	1·14, 2·12	0·005
Belief score^*^	1·09	1·05, 1·13	< 0·001
How aware are you, if at all, that utilities frequently test your water?			
Not aware at all	Reference		
Not very aware	1·26	0·77, 2·06	0·4
Somewhat aware	1·05	0·66, 1·67	0·8
Very aware	1·22	0·74, 2·03	0·4

Adjusted OR and 95 % CI from logistic regression model.

* Belief score is a composite of responses to the six water beliefs: trust in tap water, the water utility and the local government; perceived safety and quality of tap water; and awareness that the water utility frequently tests tap water.

## Discussion

In this study, we find widespread positive beliefs about the water from the faucet were not sufficient to position tap water as the preferred drinking water source, among a sample of adults living across the state of Virginia. While over two-thirds of participants had favourable ratings of trust in tap water, only half indicated a preference for tap water, and just 16 % reported a preference for unfiltered water. Our findings also indicated that trust in the water at the faucet had the strongest association with tap water preference, but perceptions of safety, quality and trust in the utility as well as the local government were also significant predictors.

A possible explanation for the preference for filtered tap water, despite the overall favourable perceptions about water directly from the faucet, is that participants filter water to improve organoleptic qualities (i.e. flavour, appearance and smell) of water; however, this was not assessed on the Water Beliefs Survey. Previous work has reported differences in preferred drinking water source even among people with similar perceptions of tap water safety^(10)^. Choosing different options at the tap (i.e. unfiltered or filtered) while having similar perceptions of water safety supports the notion that water filters are commonly used to improve taste rather than due to concerns about water safety. This suggests that trust in the safety of tap water may be necessary, but not sufficient, to motivate people to drink from the tap. Marketing of bottled water, which often positions bottled water as the purest water source, may also play a role in persuading people that bottled water is superior to the tap (i.e. healthier or safer), even if they have favourable perceptions about the water at their faucets^(7)^. The veracity of the claims that bottled water is superior to tap water has been debated, and there are several reports of bottled water producers failing inspections for maximum contaminant levels^(7,9)^. Although all types of water are at risk for contamination, water utilities typically test their water at higher rates than bottled water producers and are legally obligated to publicly report test results, which is not a requirement for bottled water producers^(7,9,16)^. Nevertheless, the volume of bottled water sold has grown exponentially over the last decade, and bottled water sales have even exceeded those of carbonated soft drinks every year since 2016^(17)^.

In the present analysis, greater education was strongly associated with a preference for tap water: Virginians with college degrees were two to three times more likely to prefer tap water compared to those who completed only high school or less. The present sample was highly educated (49 % college educated), high earning (39 % with annual incomes above 75 000) and mostly White (72 %). Having higher incomes and being non-Hispanic White have been linked to better water quality and water infrastructure^(9,18)^. These same demographics are also characteristic of those who have historically been afforded the privilege of higher education^(19)^ and effectively compounds two important precursors of trust: those who have not been systematically discriminated against will have higher trust in their government’s ability to provide safe tap water to them and those who have greater education and wealth live in better resourced communities with better tap water infrastructure. In the same vein, disinvestment in infrastructure in Black communities^(9)^ and their exclusion from educational institutions could explain the present finding that being Black was an independent predictor of lower trust in the water, the water utility and the safety of the water.

A prior analysis demonstrated that negative perceptions of water safety are associated with lower tap water use and higher bottled water use but are not associated with filtration use^(20)^. The authors of this prior study concluded that individuals who are concerned about their water safety forgo the tap altogether and consume bottled water instead, which suggests they may not perceive a filter as an effective tool for improving water safety. Although 65 % of bottled water in the USA is filtered municipal water rather than spring or well water, the ‘conventional wisdom’ in the bottled water industry is that consumers may not know that^(7)^. Opting for commercial options (e.g. bottled water) in lieu of much less expensive public services (e.g. water from the tap) has been associated with low trust in government^(9)^; and in the USA where about 85 % of the population is served by a government-owned water utility, this can have major implications on tap water consumption^(9)^. Geospatial studies linking survey data on trust in the local government with both bottled water sales and kiosk-water (private companies that market and sell ‘purified’ water in bulk) abundance have demonstrated an inverse relationship between trust and consuming these tap water alternatives^(9)^. Consistent with this literature, a direct association between trust in the government and tap water preference was observed in the present study^(9,21,22)^.

Key strengths of our study include the availability of data on water perceptions, participant characteristics and water preferences through the Water Beliefs Survey, which provided the opportunity to predict preferred water source using an adjusted statistical model. Furthermore, in addition to established determinants of tap water consumption (e.g. trust and quality), the Water Beliefs Survey included questions about awareness of water testing and trust in the government for information about water, which are relatively understudied predictors of tap water choice. To our knowledge, the Water Beliefs Survey is the largest survey on water preferences in Virginia and the first survey to separately probe trust in the water at the faucet, *v*. trust in the water utility, which had different associations with tap water preference.

Nevertheless, several limitations require consideration. First, the Water Beliefs Survey was not nationally representative and used a survey panel for participant recruitment, which limits the external generalisability of the findings. Second, the survey has not been validated to assess how tap water preference, the outcome of this study, relates to water consumption. Similarly, although people tend to consume one type of water most of the time (i.e. exclusively filtered, unfiltered or bottled)^(10)^, consumption of multiple sources of water (i.e. sometimes tap and sometimes bottled) was not captured in the Water Beliefs Survey. Further limitations are that the question about water quality did not specify whether it was asking about the water being free from contaminants or chemicals, or about organoleptic properties such as taste, smell and appearance^(21)^, nor did it consider access to chilled tap water, which is important because temperature is known to influence water palatability^(22)^. Finally, Virginia’s water systems are about 47 % publicly owned and 53 % private^(23)^; however, the present findings were not adjusted for the ownership type of the community water system. Ownership type has been associated with legal compliance of the Safe Drinking Water Act, meaning water safety violations may have affected survey participants differently^(23)^.

In summary, our results demonstrate that the majority of participants have favourable water beliefs about the water from their faucet and their water utility, but only about half consider the tap their preferred source of drinking water. This indicates that there is a gap between perceptions and consumption that future research should investigate how to narrow. These findings suggest that the utility could consider additional factors that need improvement, such as improving the palatability of the water. Furthermore, the absence of an association between awareness of the frequency of water testing and tap water preference represents a potential opportunity for the water utility to provide additional information to consumers to enhance their trustworthiness^(24,25)^. Although bottled water adds a financial burden on individuals and an environmental burden due to single-use containers and contamination from microplastics, these beverages are still highly sought after. Efforts to strengthen the trustworthiness of tap water, and its perceived quality and safety, are a critical area for future investigation with the potential to align beverage consumption with population health and environmental sustainability.

## Supporting information

Kaidbey et al. supplementary material Kaidbey et al. supplementary material

## Supplementary material

For supplementary material accompanying this paper visit https://doi.org/10.1017/S1368980025101365
