# Supplementary material for: The gap between tap water beliefs and preference for drinking from the tap: a cross-sectional study in Virginia, USA
Source: Public Health Nutr. 2025 Oct 20;28(1):e187. doi: 10.1017/S1368980025101365 (PMC12722055; doi:10.1017/S1368980025101365)
Supplement: Kaidbey et al. supplementary material [file S1368980025101365sup001.docx]

**Supplemental Table 1:** Association between self-reported water beliefs and preferred source of drinking water among 808 adults living in the state of Virginia participating in the Water Beliefs Survey.

| **Water belief** | **Chi-squared** | **p-value** |
| --- | --- | --- |
| Awareness that water utility frequently tests water | 4.91 | 0.18 |
| Trust in water utility | 19.43 | 0.0002 |
| Trust in local government as source of information | 23.34 | p< 0.0001 |
| Water safety | 38.97 | p< 0.0001 |
| Water quality | 43.67 | p< 0.0001 |
| Trust in water at the faucet | 54.28 | p< 0.0001 |

**Supplemental Table 2:** Cross-sectional associations between self-reported trust in water at the faucet and odds of preferring tap water compared to bottled water, among 808 adults living in the state of Virginia participating in the Water Beliefs Survey.

| **Characteristic** | **OR** | **95% CI** | **p-value** |
| --- | --- | --- | --- |
| How much, if at all, do you trust the water at your faucet? | | | |
| Not at all/ Not too much | Reference |  |  |
| No opinion/ Don’t know | 1.78 | 0.95, 3.39 | 0.074 |
| Some | 1.17 | 0.80, 1.72 | 0.4 |
| A lot | 3.20 | 2.06, 5.02 | <0.001 |
| Race/Ethnicity |  |  |  |
| White | Reference |  |  |
| Asian | 1.99 | 0.81, 5.28 | 0.15 |
| Black | 0.57 | 0.38, 0.86 | 0.007 |
| Hispanic or Latino | 1.12 | 0.34, 3.60 | 0.8 |
| Native American/Alaska Native | 0.81 | 0.04, 8.82 | 0.9 |
| Other/Unknown | 1.53 | 0.60, 4.15 | 0.4 |
| Gender |  |  |  |
| Female | Reference |  |  |
| Male | 0.65 | 0.46, 0.91 | 0.013 |
| Other | 1.38 | 0.57, 3.47 | 0.5 |
| Age | 1.01 | 1.00, 1.02 | 0.015 |
| Education |  |  |  |
| High school diploma or less | Reference |  |  |
| Some college or associate's degree | 1.70 | 1.10, 2.64 | 0.018 |
| College graduate or more | 2.93 | 2.02, 4.27 | <0.001 |
| Living Status |  |  |  |
| Homeowner | Reference |  |  |
| Renter | 0.73 | 0.51, 1.03 | 0.070 |
| Other | 0.47 | 0.21, 0.97 | 0.046 |
| Region of Virginia |  |  |  |
| Northern | Reference |  |  |
| Central | 1.00 | 0.59, 1.69 | >0.9 |
| Eastern | 0.69 | 0.43, 1.10 | 0.12 |
| Northwest | 1.13 | 0.65, 1.97 | 0.7 |
| Southwest | 0.75 | 0.45, 1.24 | 0.3 |
| Adjusted odds ratios (OR) and 95% confidence intervals (CI) from logistic regression model. | | | |

**Supplemental Table 3:** Cross-sectional associations between self-reported quality of water at the faucet and odds of preferring tap water compared to bottled water, among 808 adults living in the state of Virginia participating in the Water Beliefs Survey.

| **Characteristic** | **OR** | **95% CI** | **p-value** |
| --- | --- | --- | --- |
| How would you rate the quality of the water at your faucet? | | | |
| Poor | Reference |  |  |
| Just fair | 0.78 | 0.39, 1.57 | 0.5 |
| Good | 1.63 | 0.85, 3.19 | 0.15 |
| Excellent | 2.54 | 1.22, 5.36 | 0.013 |
| Race/Ethnicity |  |  |  |
| White | Reference |  |  |
| Asian | 1.92 | 0.79, 5.08 | 0.2 |
| Black | 0.58 | 0.38, 0.87 | 0.009 |
| Hispanic or Latino | 1.28 | 0.39, 4.01 | 0.7 |
| Native American/Alaska Native | 0.69 | 0.03, 7.86 | 0.8 |
| Other/Unknown | 1.38 | 0.55, 3.67 | 0.5 |
| Gender |  |  |  |
| Female | Reference |  |  |
| Male | 0.69 | 0.50, 0.97 | 0.032 |
| Other | 1.23 | 0.51, 3.13 | 0.7 |
| Age | 1.02 | 1.01, 1.03 | 0.002 |
| Education |  |  |  |
| High school diploma or less | Reference |  |  |
| Some college or associate's degree | 1.80 | 1.16, 2.80 | 0.009 |
| College graduate or more | 3.13 | 2.16, 4.55 | <0.001 |
| Living Status |  |  |  |
| Homeowner | Reference |  |  |
| Renter | 0.77 | 0.55, 1.09 | 0.15 |
| Other | 0.47 | 0.22, 0.98 | 0.047 |
| Region of Virginia |  |  |  |
| Northern | Reference |  |  |
| Central | 1.04 | 0.62, 1.75 | 0.9 |
| Eastern | 0.74 | 0.46, 1.19 | 0.2 |
| Northwest | 1.18 | 0.68, 2.05 | 0.6 |
| Southwest | 0.78 | 0.47, 1.29 | 0.3 |
| Adjusted odds ratios (OR) and 95% confidence intervals (CI) from logistic regression model. | | | |

**Supplemental Table 4:** Cross-sectional associations between self-reported safety of water at the faucet and odds of preferring tap water compared to bottled water, among 808 adults living in the state of Virginia participating in the Water Beliefs Survey.

| **Characteristic** | **OR** | **95% CI** | **p-value** |
| --- | --- | --- | --- |
| In your view, how safe or unsafe is the water at your faucet? |  |  |  |
| Not safe at all/ not very safe | Reference |  |  |
| Somewhat unsafe | 0.95 | 0.53, 1.70 | 0.9 |
| Somewhat safe | 1.30 | 0.82, 2.06 | 0.3 |
| Very safe | 2.32 | 1.42, 3.82 | <0.001 |
| Race/Ethnicity |  |  |  |
| White | Reference |  |  |
| Asian | 1.84 | 0.77, 4.77 | 0.2 |
| Black | 0.57 | 0.38, 0.86 | 0.008 |
| Hispanic or Latino | 1.12 | 0.35, 3.50 | 0.8 |
| Native American/Alaska Native | 0.73 | 0.03, 8.01 | 0.8 |
| Other/Unknown | 1.49 | 0.59, 3.97 | 0.4 |
| Gender |  |  |  |
| Female | Reference |  |  |
| Male | 0.69 | 0.49, 0.96 | 0.027 |
| Other | 1.41 | 0.59, 3.52 | 0.5 |
| Age | 1.01 | 1.00, 1.02 | 0.004 |
| Education |  |  |  |
| High school diploma or less | Reference |  |  |
| Some college or associate's degree | 1.70 | 1.10, 2.65 | 0.017 |
| College graduate or more | 2.93 | 2.02, 4.27 | <0.001 |
| Living Status |  |  |  |
| Homeowner | Reference |  |  |
| Renter | 0.75 | 0.53, 1.06 | 0.10 |
| Other | 0.47 | 0.22, 0.97 | 0.044 |
| Region of Virginia |  |  |  |
| Northern | Reference |  |  |
| Central | 1.03 | 0.62, 1.73 | >0.9 |
| Eastern | 0.71 | 0.44, 1.13 | 0.15 |
| Northwest | 1.18 | 0.68, 2.04 | 0.6 |
| Southwest | 0.77 | 0.47, 1.27 | 0.3 |
| Adjusted odds ratios (OR) and 95% confidence intervals (CI) from logistic regression model. | | | |

**Supplemental Table 5:** Cross-sectional associations between self-reported trust in the water utility and odds of preferring tap water compared to bottled water, among 808 adults living in the state of Virginia participating in the Water Beliefs Survey.

| **Characteristic** | **OR** | **95% CI** | **p-value** |
| --- | --- | --- | --- |
| How much, if at all, do you trust your water utility company? | | | |
| Not at all/ Not too much | Reference |  |  |
| No opinion/ Don’t know | 1.33 | 0.70, 2.55 | 0.4 |
| Some | 1.33 | 0.78, 2.31 | 0.3 |
| A lot | 1.86 | 1.06, 3.28 | 0.030 |
| Race/Ethnicity |  |  |  |
| White | Reference |  |  |
| Asian | 1.70 | 0.71, 4.45 | 0.2 |
| Black | 0.55 | 0.37, 0.83 | 0.004 |
| Hispanic or Latino | 0.98 | 0.30, 3.06 | >0.9 |
| Native American/Alaska Native | 0.61 | 0.03, 6.61 | 0.7 |
| Other/Unknown | 1.40 | 0.56, 3.74 | 0.5 |
| Gender |  |  |  |
| Female | Reference |  |  |
| Male | 0.72 | 0.51, 1.00 | 0.049 |
| Other | 1.36 | 0.57, 3.37 | 0.5 |
| Age | 1.01 | 1.01, 1.02 | 0.002 |
| Education |  |  |  |
| High school diploma or less | Reference |  |  |
| Some college or associate's degree | 1.69 | 1.10, 2.61 | 0.017 |
| College graduate or more | 3.05 | 2.11, 4.42 | <0.001 |
| Living Status |  |  |  |
| Homeowner | Reference |  |  |
| Renter | 0.74 | 0.53, 1.04 | 0.086 |
| Other | 0.47 | 0.22, 0.97 | 0.044 |
| Region of Virginia |  |  |  |
| Northern | Reference |  |  |
| Central | 1.05 | 0.63, 1.74 | 0.9 |
| Eastern | 0.74 | 0.47, 1.18 | 0.2 |
| Northwest | 1.19 | 0.69, 2.05 | 0.5 |
| Southwest | 0.76 | 0.46, 1.25 | 0.3 |
| Adjusted odds ratios (OR) and 95% confidence intervals (CI) from logistic regression model. | | | |

**Supplemental Table 6:** Cross-sectional associations between considering the local government as a trusted source of information about water and odds of preferring tap water compared to bottled water, among 808 adults living in the state of Virginia participating in the Water Beliefs Survey.

| **Characteristic** | **OR** | **95% CI** | **p-value** |
| --- | --- | --- | --- |
| Local government is a trusted source of information (yes) | 1.56 | 1.14, 2.12 | 0.005 |
| Race/Ethnicity |  |  |  |
| White | Reference |  |  |
| Asian | 1.64 | 0.68, 4.28 | 0.3 |
| Black | 0.55 | 0.36, 0.82 | 0.004 |
| Hispanic or Latino | 0.91 | 0.28, 2.85 | 0.9 |
| Native American/Alaska Native | 0.54 | 0.02, 6.06 | 0.6 |
| Other/Unknown | 1.52 | 0.61, 4.08 | 0.4 |
| Gender |  |  |  |
| Female | Reference |  |  |
| Male | 0.73 | 0.53, 1.01 | 0.061 |
| Other | 1.34 | 0.56, 3.36 | 0.5 |
| Age | 1.01 | 1.00, 1.02 | 0.006 |
| Education |  |  |  |
| High school diploma or less | Reference |  |  |
| Some college or associate's degree | 1.67 | 1.09, 2.57 | 0.020 |
| College graduate or more | 2.94 | 2.04, 4.27 | <0.001 |
| Living Status |  |  |  |
| Homeowner | Reference |  |  |
| Renter | 0.72 | 0.51, 1.01 | 0.057 |
| Other | 0.45 | 0.21, 0.92 | 0.032 |
| Region of Virginia |  |  |  |
| Northern | Reference |  |  |
| Central | 1.04 | 0.62, 1.73 | 0.9 |
| Eastern | 0.75 | 0.47, 1.19 | 0.2 |
| Northwest | 1.25 | 0.73, 2.16 | 0.4 |
| Southwest | 0.76 | 0.46, 1.25 | 0.3 |
| Adjusted odds ratios (OR) and 95% confidence intervals (CI) from logistic regression model. | | | |

**Supplemental Table 7:** Cross-sectional associations between self-reported awareness of the frequency of water testing and odds of preferring tap water compared to bottled water, among 808 adults living in the state of Virginia participating in the Water Beliefs Survey.

| **Characteristic** | **OR** | **95% CI** | **p-value** |
| --- | --- | --- | --- |
| How aware are you, if at all, that utilities frequently test your water? | | | |
| Not aware at all | Reference |  |  |
| Not very aware | 1.26 | 0.77, 2.06 | 0.4 |
| Somewhat aware | 1.05 | 0.66, 1.67 | 0.8 |
| Very aware | 1.22 | 0.74, 2.03 | 0.4 |
| Race/Ethnicity |  |  |  |
| White | Reference |  |  |
| Asian | 1.72 | 0.71, 4.51 | 0.2 |
| Black | 0.55 | 0.36, 0.82 | 0.004 |
| Hispanic or Latino | 0.94 | 0.29, 2.94 | >0.9 |
| Native American/Alaska Native | 0.58 | 0.03, 6.33 | 0.7 |
| Other/Unknown | 1.41 | 0.57, 3.78 | 0.5 |
| Gender |  |  |  |
| Female | Reference |  |  |
| Male | 0.75 | 0.54, 1.04 | 0.09 |
| Other | 1.40 | 0.59, 3.49 | 0.5 |
| Age | 1.02 | 1.01, 1.03 | 0.001 |
| Education |  |  |  |
| High school diploma or less | Reference |  |  |
| Some college or associate's degree | 1.64 | 1.06, 2.52 | 0.02 |
| College graduate or more | 3.13 | 2.17, 4.53 | <0.001 |
| Living Status |  |  |  |
| Homeowner | Reference |  |  |
| Renter | 0.72 | 0.51, 1.02 | 0.06 |
| Other | 0.44 | 0.21, 0.89 | 0.03 |
| Region of Virginia |  |  |  |
| Northern | Reference |  |  |
| Central | 1.03 | 0.62, 1.71 | >0.9 |
| Eastern | 0.72 | 0.46, 1.14 | 0.2 |
| Northwest | 1.17 | 0.68, 2.02 | 0.6 |
| Southwest | 0.72 | 0.44, 1.19 | 0.2 |
| Adjusted odds ratios (OR) and 95% confidence intervals (CI) from logistic regression model. | | | |

**Supplemental Table 8:** Cross-sectional associations between composite belief score and odds of preferring tap water compared to bottled water, among 808 adults living in the state of Virginia surveyed in the Water Beliefs Survey.

| **Characteristic** | **OR** | **95% CI** | **p-value** |
| --- | --- | --- | --- |
| Belief score^a^ | 1.09 | 1.05, 1.13 | <0.001 |
| Race/Ethnicity |  |  |  |
| White | Reference |  |  |
| Asian | 1.79 | 0.74, 4.71 | 0.2 |
| Black | 0.57 | 0.38, 0.85 | 0.006 |
| Hispanic or Latino | 1.09 | 0.33, 3.43 | 0.9 |
| Native American/Alaska Native | 0.57 | 0.03, 6.48 | 0.7 |
| Other/Unknown | 1.60 | 0.64, 4.30 | 0.3 |
| Gender |  |  |  |
| Female | Reference |  |  |
| Male | 0.66 | 0.47, 0.92 | 0.016 |
| Other | 1.25 | 0.52, 3.13 | 0.6 |
| Age | 1.01 | 1.00, 1.02 | 0.008 |
| Education |  |  |  |
| High school diploma or less | Reference |  |  |
| Some college or associate's degree | 1.71 | 1.11, 2.66 | 0.016 |
| College graduate or more | 2.86 | 1.97, 4.16 | <0.001 |
| Living Status |  |  |  |
| Homeowner | Reference |  |  |
| Renter | 0.77 | 0.55, 1.09 | 0.14 |
| Other | 0.49 | 0.23, 1.00 | 0.056 |
| Region of Virginia |  |  |  |
| Northern | Reference |  |  |
| Central | 1.05 | 0.63, 1.76 | 0.9 |
| Eastern | 0.76 | 0.47, 1.20 | 0.2 |
| Northwest | 1.24 | 0.72, 2.15 | 0.4 |
| Southwest | 0.82 | 0.49, 1.36 | 0.4 |
| Adjusted odds ratios (OR) and 95% confidence intervals (CI) from logistic regression model.  ^a^ Belief score is a composite of response ratings to six self-reported beliefs about tap water: trust in tap water, the water utility provider, and the local government; perceived safety and quality of tap water; and awareness that the water utility frequently tests tap water. | | | |
|  | | | |

**Supplemental Table 9**: Association between water beliefs and preference for unfiltered tap water compared to filtered tap water or bottled water, among 808 adults living in the state of Virginia that participated in the Water Beliefs Survey.

| **Multivariable model independent variable** | **OR** | **95% CI** | **p-value** |
| --- | --- | --- | --- |
| **How much, if at all, do you trust the water at your faucet?** |  |  |  |
| Not at all/ Not too much | Reference |  |  |
| No opinion/ Don’t know | 9.12 | 2.83, 32.9 | <0.001 |
| Some | 4.81 | 1.95, 14.6 | 0.002 |
| A lot | 29.4 | 11.8, 90.0 | <0.001 |
| **How would you rate the quality of the water at your faucet?** |  |  |  |
| Poor | Reference |  |  |
| Just fair | 0.70 | 0.19, 3.40 | 0.6 |
| Good | 2.98 | 0.94, 13.3 | 0.10 |
| Excellent | 12.6 | 3.71, 59.0 | <0.001 |
| **In your view, how safe or unsafe is the water at your faucet?** |  |  |  |
| Not safe at all/ not very safe | Reference |  |  |
| Somewhat unsafe | 2.62 | 0.77, 10.5 | 0.14 |
| Somewhat safe | 4.01 | 1.47, 14.2 | 0.014 |
| Very safe | 17.1 | 6.26, 61.0 | <0.001 |
| **How much, if at all, do you trust your water utility company?** |  |  |  |
| Not at all/ Not too much | Reference |  |  |
| No opinion/ Don’t know | 1.20 | 0.36, 4.41 | 0.8 |
| Some | 2.26 | 0.88,7.05 | 0.12 |
| A lot | 5.28 | 2.04, 16.6 | 0.002 |
| **Local government is a trusted source of information (yes)** | 2.02 | 1.28, 3.21 | 0.003 |
| **Belief score** ^a^ | 1.30 | 1.21, 1.40 | <0.001 |
| **How aware are you, if at all, that utilities frequently test your water?** |  |  |  |
| Not aware at all | Reference |  |  |
| Not very aware | 1.85 | 0.86, 4.19 | 0.12 |
| Somewhat aware | 1.65 | 0.80, 3.60 | 0.2 |
| Very aware | 2.01 | 0.92, 4.62 | 0.087 |
| Adjusted odds ratios (OR) and 95% confidence intervals (CI) from logistic regression model.  ^a^ Belief score is a composite of response ratings to six self-reported beliefs about tap water: trust in tap water, the water utility provider, and the local government; perceived safety and quality of tap water; and awareness that the water utility frequently tests tap water. | | | |
